# Interleukin-13 as a target to alleviate severe coronavirus disease 2019 and restore lung homeostasis

**Published:** 2021-01-27

**Authors:** Lachlan Paul Deimel, Zheyi Li, Charani Ranasinghe

**Affiliations:** Molecular Mucosal Vaccine Immunology Group, Department of Immunology and Infectious Disease, The John Curtin School of Medical Research, The Australian National University, Canberra ACT 2601, Australia

**Keywords:** coronavirus disease-19, severe acute respiratory syndrome coronavirus 2, interleukin-13, lung mucosae, ILC2, inflammation, cytokine storm, interleukin-4/interleukin-13 antagonists, IL-13Ra2

## Abstract

The ongoing coronavirus disease (COVID-19) pandemic urgently requires the availability of interventions that improve outcomes for those with severe disease. Since severe acute respiratory syndrome coronavirus 2 infection is characterized by dysregulated lung mucosae, and that mucosal homeostasis is heavily influenced by interleukin (IL)-13 activity, we explore recent findings indicating that IL-13 production is proportional to disease severity. We propose that excessive IL-13 contributes to the progression of severe/fatal COVID-19 by (1) promoting the recruitment of immune cells that express inflammatory cytokines, causing a cytokine storm that results in widespread destruction of lung tissue, (2) directly facilitating tissue-remodeling that causes airway hyperinflammation and obstruction, and (3) diverting the immune system away from developing high-quality cytotoxic T cells that confer effective anti-viral immunity. These factors may cumulatively result in significant lung distress, multi-organ failure, and death. Here, we suggest repurposing existing IL-13-inhibiting interventions, including antibody therapies routinely used for allergic lung hyperinflammation, as well as viral vector-based approaches, to alleviate disease. Since many of these strategies have previously been shown to be both safe and effective, this could prove to be a highly cost-effective solution.

**Relevance for Patients:**

There remains a desperate need to establish medical interventions that reliably improves outcomes for patients suffering from COVID-19. We explore the role of IL-13 in maintaining homeostasis at the lung mucosae and propose that its dysregulation during viral infection may propagate the hallmarks of severe disease – further exploration may provide a platform for invaluable therapeutics.

Interleukin (IL)-13 is critical in maintaining mucosal homeostasis, being implicated in allergy, parasitic and viral infection, as well as vaccine-specific immunity [1-8]. At the lung mucosae, IL-13 is expressed by a range of innate immune cell types, particularly type 2 innate lymphoid cells (ILC2s), whose rapid response to external stimuli (pathogens, toxins, and allergens) acts to facilitate barrier tissue responses and condition downstream immune outcomes [9-12]. IL-13 activity at the lung triggers smooth muscle contraction, mucus secretion, and the recruitment/activation of inflammatory immune cells. However, overexpression of IL-13 is associated with allergic lung hyperinflammation, airway tissue remodeling, and hyperresponsiveness [1,13-15]. Interestingly, IL-13 dysregulation is known to be a hallmark of several disease conditions, including allergic pulmonary diseases, atopic dermatitis, and also some cancers [16-19]. Since coronavirus disease 2019 (COVID-19) is fundamentally characterized by dysregulation of the lung mucosae, we postulate that IL-13 is associated with destructive lung hyperinflammation/immune activity that underpins severe COVID-19 disease progression. Here we discuss how IL-13-inhibiting interventions could be repurposed to benefit severe acute respiratory syndrome coronavirus 2 (SARS-CoV2)-infected patients.


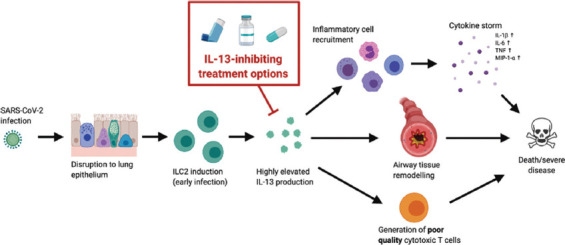


By studying a series of viruses, we have recently shown that ILC2s produce significant IL-13 following viral infection/vaccination 24 h post-encounter [10], where the level of ILC2-derived IL-13 is dependent on the virus (e.g., fowlpox <influenza <rhinovirus <*Vaccinia virus*) [11,20]. Moreover, at the later stages of viral infection, Th2 cells can also contribute to the IL-13 environment, impacting the resulting viral load and adaptive immune outcomes [3,21,22]. Interestingly, dramatically elevated IL-13 levels have been reported at the lung mucosae in SARS-CoV-2-infected individuals [23]. Therefore, we suspect that COVID-19 patients may display significant lung ILC2-derived IL-13 (although the role of ILC2s during SARS-CoV-2 infection is yet to be fully realized) [24,25]. Even though some IL-13 can be helpful during respiratory viral infection by aiding effective antibody differentiation [26,27], recruitment of different immune cells [28-30], and coordination of amphiregulin-dependent epithelium restoration [31], excessive production is damaging to airway homeostasis. Excessive IL-13 at the lung mucosae could be a key determinant of COVID-19-related hyper-inflammation [23]. Donlan *et al*. have recently shown that IL-13 levels are a powerful predictor of COVID-19 severity and the need for ventilation, independently of age, gender, and comorbidity [32]. This is unsurprising, given that many characteristics of fatal disease can be attributed to symptoms of dysregulated IL-13 [33]. Moreover, it is also noteworthy that the production of elevated Th2 cytokines, IL-4, and IL-13 is thought to be an inherent mechanism by which viruses evade the host immune system, promoting the induction of poor-quality cytotoxic T cell immunity [3,34-36].

It is well-established that IL-13 can effectively recruit inflammatory neutrophils, macrophages, eosinophils, and lymphocytes to the lung mucosae, resulting in elevated expression of various pro-inflammatory cytokines/chemokines [14,15,37-40]. Interestingly, patients with severe COVID-19 have shown to overexpress cytokines IL-1β, IL-6, tumor necrosis factor, and macrophage inflammatory protein-1-a, which can inadvertently promote overwhelming tissue damage [33,41,42]. The hyper-inflammatory phenotype and underlying cytokine storm is thought to be the primary cause of COVID-19-associated death, resulting in acute respiratory distress syndrome and subsequent multi-organ failure [43]. Collectively, these observations indicate that IL-13 may underpin inflammatory immune cell representations at the lung to drive cytokine storming in patients with COVID-19.

Further, IL-13 is well-known to have direct implications on lung tissue remodeling, airway obstruction, and acute/chronic lung damage in both allergy and chronic obstructive pulmonary disease [44]. Specifically, IL-13 facilitates airway smooth muscle proliferation, fibroblast proliferation, goblet cell hyperplasia, parenchymal inflammation, and collagen deposition [13,14,45-47], many of which have been observed in patients with fatal COVID-19 [33]. Thus, we suspect that IL-13 may be the upstream mediator of severe SARS-CoV-2 disease.

Moreover, IL-33 is a key upstream mediator of IL-13 at the lung mucosae and is thought to play a role in COVID-19 pathogenesis [48]. IL-33 is an alarmin produced by epithelial cells/alveolar macrophages to recruit and activate immune cells, particularly IL-33R^+^ lung ILC2s [49]. Interestingly, our recent studies have shown that transient sequestration of IL-33 at the lung mucosae using a viral vector expressing IL-33RBP (binding protein) does not impact ILC2-drived IL-13 expression. In contrast, IL-25RBP has a marked impact on ILC2-derived IL-13 [50]. This indicates a complex hierarchy between these cytokines. Notably, other studies have also shown that IL-33, IL-25, and thymic stromal lymphopoietin differentially modulate ILC2 activity, specifically in the context of tissue remodeling, allergy, and inflammation [51,52]. However, we propose that in the context of alleviating severe COVID-19, direct inhibition of IL-13 may yield better disease outcomes rather than targeting a particular upstream determinant of IL-13 expression.

In comparative respiratory conditions with similar molecular and immunological signatures, restricting IL-13 signaling has improved patient outcomes. For example, treatment with a monoclonal human anti-IL-4Rα antibody dupilumab (which inhibits both IL-13 and IL-4 signaling) has shown significant benefits in patients with otherwise uncontrollable asthma or severe dermatitis [53,54]. Interestingly, it has been proposed that such interventions could be unfavorable in treating COVID-19, in part due to the Th1/Th17 cytokines involved in hyperinflammation, where IL-13/IL-4 inhibition may further bias in immune activity [55]. However, our laboratory and others have demonstrated that IL-13 does not necessarily adhere to the classical Th1/Th2 immune paradigm, as exemplified by the broad profile of immune cells it modulates and/or recruits [3,14,20,48,56,57]. Importantly, Dupilumab, along with its favorable safety profile, is widely known to reduce airway inflammation (including Th1/Th17 cytokines) and improve global lung function (such as improve forced expiration volume) [53,58-60]. Similar findings have also been reported in asthmatics using Tralokinumab, which directly binds to and neutralizes IL-13. However, while Tralokinumab clearly improves spirometric outputs, limited benefit to quality of life has been reported [61,62]. Thus, at early stages of SARS-CoV-2 viral infection, IL-13 inhibition at the lung mucosae may help reduce COVID-19 disease severity/progression.

Alternatively, viral vectors have long been utilized as vehicles to express vaccine antigens, immunomodulators, cytokines/chemokines, and cytokine receptors [63,64]. We have studied the use of viral vectors that co-express vaccine antigens with either (1) mutant IL-4 lacking the signaling domain that can bind to and antagonize IL-4Rα to restrict the signaling of STAT6 or (2) IL-13Rα2 that sequesters excess IL-13 at the vaccination site to improve the quality of cytotoxic T cell immunity [20,22,27]. In the context of COVID-19, a viral vector-based approach to transiently inhibit excess IL-13 at the lung mucosae may help alleviate severe disease similarly to therapies using monoclonal antibodies. However, an attenuated viral vector could be a more attractive approach, with a single dose offering long lasting (~3 days) benefit, while still being safe and providing a highly localized/targeted response. However, in this context, selecting a viral vector that induces low IL-13 would be of great importance, as vectors themselves can promote the induction of ILC2-derived IL-13 and DC activity at the lung mucosae [11,50,57]

In conclusion, knowing that IL-13 is a powerful indicator of COVID-19 severity [14,32], interventions that directly inhibit IL-13 activity at the lung mucosae may prove useful in preventing or reducing disease progression. Since safe and effective IL-13 inhibiting drugs/therapies are already available (such as allergy/asthma treatments and recombinant viral vectors) [53,58], their repurposing could be a highly cost-effective solution in alleviating SARS-CoV-2-associated pathology. This warrants investigation.
